# Efficacy of stem cells on the healing of peri‐implant defects: systematic review of preclinical studies

**DOI:** 10.1002/cre2.16

**Published:** 2016-02-04

**Authors:** Mônica Yuri Orita Misawa, Guy Huynh‐Ba, Gustavo Machado Villar, Cristina Cunha Villar

**Affiliations:** ^1^ Division of Periodontics, Department of Stomatology, School of Dentistry University of São Paulo São Paulo São Paulo Brazil; ^2^ Department of Periodontics University of Texas Health Science Center at San Antonio Dental School San Antonio Texas USA

**Keywords:** Animal experimentation, bone formation, dental implants, mesenchymal stem cells, systematic review

## Abstract

This systematic review considers the evidence from animal studies evaluating the effectiveness of mesenchymal stem cells (MSC) in the treatment of intraoral peri‐implant defects. MEDLINE, EMBASE, and LILACS databases were searched for quantitative preclinical controlled animal model studies that evaluated the effect of MSC on bone healing at intraoral peri‐implant bone defects. The primary outcome was the amount of (re‐)osseointegration reported as bone‐to‐implant contact in the defect area. The systematic review was conducted according to the Preferred Reporting Items for Systematic Reviews and Meta‐Analyses statement guidelines. Ten studies met the inclusion criteria. Only one study induced peri‐implant inflammation to produce peri‐implant bone defects. In all others, defects were surgically created at implant installation. Differences in defect morphology were identified among the studies. Both xenogenous and autogenous MSC were used to treat peri‐implant defects. These included bone marrow‐derived MSC, periodontal ligament‐derived MSC, umbilical cord MSC, bone marrow‐derived mononuclear cells, and peripheral blood mononuclear cells. Meta‐analysis was not possible because of heterogeneities in study designs. Nonetheless, in most studies, local MSC implantation was not associated with adverse effects and had a positive effect on bone healing around peri‐implant defects. Combination of MSC with membranes and bioactive factors appears to provide improved treatment outcomes. In large animal models, intraoral use of MSC may provide beneficial effects on bone healing within peri‐implant defects. The various degrees of success of MSC in peri‐implant bone healing are likely to be related to the use of cells from various populations, tissues, and donor species. However, human safety and efficacy must be demonstrated before its clinical use can be considered.

## Introduction

Dental implant therapy is a well‐accepted treatment modality among clinicians and academicians to replace missing teeth (Mertens et al. 2012; Östman et al. 2012; Pjetursson et al. 2005). The prerequisite for implant success is, on the short term, the presence of direct bone‐to‐implant contact (BIC) following healing and, on the long term, maintenance of osseointegration with minimal bone loss over time (Albrektsson et al. 1986). Initially, it was suggested that implants should be placed in healed ridges with adequate amounts of bone. Developments over time have allowed to successfully address more challenging clinical situations where insufficient amount of bone is present. Such situations may be represented by bone defects around immediate implants (Capelli et al. 2013, Vignoletti and Sanz 2014), insufficient alveolar ridge width (Benic and Hämmerle 2014, Wachtel et al. 1991), and defects resulting from peri‐implantitis lesions (Heitz‐Mayfield and Mombelli 2014).

While different bone grafting materials have been implemented in the treatment of the aforementioned peri‐implant bone defects, autogenous grafts have the unique ability to form bone by osteogenesis, osteoinductivity, and osteoconductivity and may therefore be considered as a gold standard for bone regeneration (Kao and Scott 2007). However, this advantage is counterbalanced by the limited amounts that may be available for harvesting and need for a second surgical site that is associated with potential patient morbidities (Nkenke and Neukam 2014). Alternatives including allografts, xenografts, and alloplastic material have demonstrated only limited regenerative potential at best. Therefore, an ideal treatment alternative would overcome the shortcomings of autogenous grafts while maintaining similar regenerative properties. Such an alternative may be represented by tissue‐engineering therapies.

Tissue‐engineering therapy is widely applied in the medical and dental fields to regenerate the function of lost or damaged tissue. To achieve this goal tissue‐engineering strategies rely on a triad, which encompasses cells with regenerative potential (i.e., stem cells), signaling molecules such as growth factors, and a biocompatible matrix serving as a scaffold (Langer and Vacanti 1993). Recent advances in tissue‐engineering strategies led to the development of living cell‐based therapies to regenerate lost or damaged tissues, including myocardial tissue (Genovese et al. 2007), long bones (Bueno and Glowacki 2009), and skin (El‐Mesallamy et al. 2014). In the dental field, reconstruction of the craniofacial skeleton and the temporomandibular joint (Shanti et al. 2007), regeneration of the pulpal (Hargreaves et al. 2013), and periodontal tissues (Hynes et al. 2012; Pejcic et al. 2013) and bone regeneration (Nishimura et al. 2012) illustrate a few applications of cell‐based therapies. Mesenchymal stem cells (MSC) are non‐hematopoietic progenitor cells that have the ability to differentiate into distinct mesenchymal cell lineages, including into osteoblastic lineages. Accordingly, MSC represent a promising alternative to bone grafts in the treatment of intraoral peri‐implant defects, and its effectiveness will be evaluated in the present systematic review.

## Material and Methods

### Focused question

We conducted a systematic review of the literature to address the following focused Patient, Intervation, Control, Outcome (PICO) question: “In animal models, do mesenchymal stem cells improve bone healing at intraoral peri‐implant bone defects, as compared to controls?”

This systematic review was conducted according to the Preferred Reporting Items for Systematic Reviews and Meta‐analyses statement guidelines (Liberati et al. 2009).

### Eligibility criteria

#### Type of studies

Only preclinical controlled animal model studies using MSC for the treatment of intraoral peri‐implant bone defects were eligible for this review.

### Study population

The population of interest for this review included large animals with no systemic conditions or genetic modifications.

#### Type of intervention and type of comparison

Treatment of intraoral peri‐implant bone defects using MSC (test group) was compared with control treatments. The protocol of control groups varied according to the type of intervention in each study.

#### Outcome measures

The primary outcome was the amount of (re‐)osseointegration of the implant reported as BIC in the defect area. The secondary outcome variable was new bone formation within peri‐implant defects.

### Search strategy

Search strategies were developed for MEDLINE, EMBASE, and LILACS databases. Medical subject headings terms and keywords were combined with Boolean operators and used to search the databases. All searches were performed without language restriction, up to March 2015. The following keywords and medical subject headings terms were used ((((stem cells OR mesenchymal dental cells OR mesenchymal dental follicle OR mesenchymal dental papilla OR mesenchymal dental stem OR mesenchymal derived OR mesenchymal differentiation OR mesenchymal epithelial OR mesenchymal fibroblast OR mesenchymal fibroblastic OR mesenchymal like OR mesenchymal lineage OR mesenchymal multipotent OR mesenchymal odontoblasts OR mesenchymal origin OR mesenchymal papilla OR mesenchymal soft tissue OR mesenchymal stem OR mesenchymal stem/precursor cells OR mesenchymal stem/progenitor cells OR mesenchymal stem/stromal cells OR mesenchymal stem cell OR mesenchymal stem cell derived OR mesenchymal stem cell like OR mesenchymal stem cell MSC OR mesenchymal stem cell MSCS OR mesenchymal stromal cells OR dental pulp stromal OR periodontal ligament like OR periodontal ligament stem OR periodontal ligament stromal cells OR periodontal mesenchymal cells OR periodontal ligament progenitor OR periodontal ligament cells OR gingival margin derived cell OR periapical follicle cells OR dental follicular cells OR dental follicle cells OR dental follicle precursor cells OR dental follicle progenitor cells OR dental follicle stem OR oral mucosa stem cells OR bone marrow cells OR bone marrow derived OR bone marrow stem OR bone marrow stroma OR IPS cell OR adipose mesenchymal stem OR adipose MSCS OR adipose progenitor cells OR adipose stem cell OR adipose stroma OR adipose stromal cells OR pluripotent cells OR multipotent cells)) AND (peri‐implant defects OR peri‐implant defects OR peri‐implantitis OR peri‐implantitis OR dental implants OR implant infection OR implant bone defect)) NOT “clinical trial”) NOT review. Manual searches of reference lists from selected full articles complemented the electronic search.

### Exclusion criteria

Reviews, *in vitro* and human studies, and animal studies without control groups were excluded.

### Screening methods and data extraction

Two calibrated reviewers (C. C. V. and G. H.) screened independently titles and abstracts. Studies appearing to meet the inclusion criteria, or those with insufficient information in the title and abstract to make a clear decision, were selected for evaluation of the full manuscript, which was carried independently by the same two reviewers to determine study eligibility. Any disagreement was solved by discussion and agreement between the reviewers. All studies that met the inclusion criteria underwent a validity assessment. Reasons for rejecting studies were recorded for each study. Agreement between reviewers was described by kappa coefficient. Data were extracted independently by two reviewers (C. C. V. and G. M. V.), with disagreements resolved by discussion with a third reviewer (M. Y. O. M.). Authors of six publications were contacted to clarify data or to provide missing information (Park et al. 2014; Yun et al. 2014; Ribeiro et al. 2012; Wang et al. 2011; Kim et al. 2009; Ito et al. 2006).

The following data were extracted and recorded: citation, MSC origin, stem cell characterization, animal model, number of animals, number of defects per group, defect type and size, location of the defect, treatment, and length of follow‐up.

### Quality assessment and data synthesis

Quality assessment of included studies was performed independently by two reviewers (C. C. V. and G. M. V.), blinded to the name of the authors, institutions, and journal titles. Any disagreements were solved by discussion with a third reviewer (M. Y. O. M.). The following six domains were assessed as having “low risk,” “high risk,” or “unclear risk” of bias, according to the Cochrane Collaboration's tool for assessing risk of bias (Higgins et al. 2011).

- Selection bias
  - Random sequence generation
  - Allocation concealment
- Performance bias
  - Blinding of participants and personnel
- Detection bias
  - Blinding of outcome assessor
- Attrition bias
  - Incomplete outcome data
- Reporting bias
  - Selective reporting
- Other bias
  - Other sources of bias (related to the design and conduct of the trial, precision, reporting standards, and ethical criteria).

Using the Cochrane's Risk of Bias tool, included studies were categorized as follows: (1) “low‐risk” of bias (plausible bias unlikely to seriously alter the results), if all domains were met; (2) “unclear risk” of bias (plausible bias that raises some doubt about the results), if one or more domains were classified as having unclear risk of bias; and (3) “high risk” of bias (plausible bias seriously weakens the confidence in the results), if one or more domains were not met.

## Results

**Figure 1:**
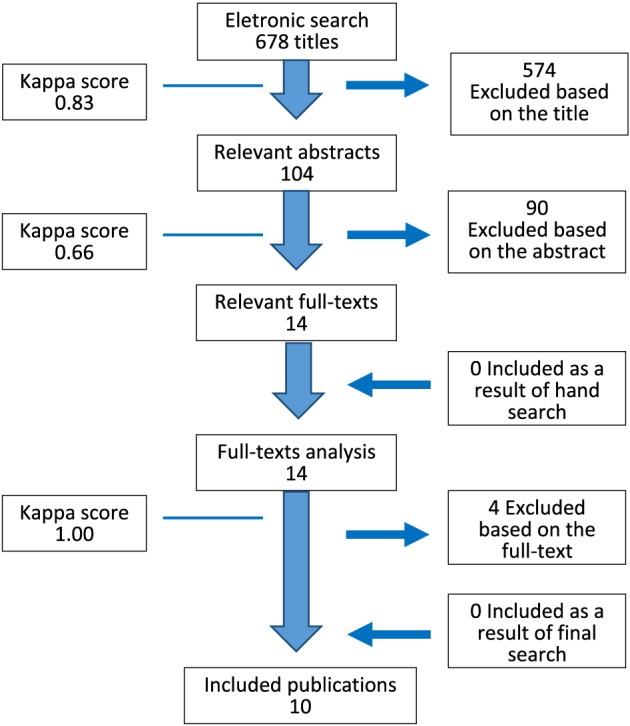
Simplified search strategy outline.

The computerized search strategy yielded 678 citations, of which, 104 were screened for potentially meeting the inclusion criteria (*κ* = 0.83; Fig. 1). Independent screening of abstracts led to the rejection of 90 articles (*κ* = 0.66; Fig. 1). Full texts of the remaining 14 publications were obtained for review and possible inclusion in the systematic review and meta‐analysis. Out of these, four articles were further excluded (*κ* = 1.00) (Hao et al. 2014a; Hoşgör et al. 2013; Zhang et al. 2007; Ribeiro et al. 2010) for reasons indicated in Table 1. Manual search of reference lists of selected studies yielded no additional articles (Fig. 1). Characteristics of the final 10 retained studies are reported in Table 2.

**Table 1:** Excluded studies.

Study	Reason for exclusion
Hao et al. (2014a)	Repeated article.
Hoşgör et al. (2013)	Implants were installed in extraoral sites
Ribeiro et al. (2010)	The study lacked a negative control without cells; two experimental groups received BM‐MSC and PDLSC, respectively
Zhang (2007)	None of the experimental groups evaluated the treatment of peri‐implant defects with MSC

MSC, mesenchymal stem cells; BM‐MSC, bone marrow‐derived mesenchymal stem cells; PDLSC, periodontal ligament‐derived mesenchymal stem cells.

**Table 2:** Details of the studies included.

Study	MSC origin (*n*)/ characterization/ state of differentiation at application	Animal model/*n* (*n* of defects)	Defect type/size	Location of defect	Treatment Groups	Observational period	Results
Xu et al. (2015)	Autogenous iliac crest BM‐MSC (*n* = 6). Colony‐forming efficiency. Undifferentiated at application	6 adult male Labrador dogs (24 defects)	Surgically created three‐wall intrabony defects at the mesial aspect of the mesial extraction sockets (6 × 4 × 5 mm)	P1‐P4 mesial extraction socket	Group 1: βTCP (*n* = 6)	3 months	BIC (%) in the midportion of the defect
					Group 2: βTCP + rhPDGF‐BB (*n* = 6)		βTCP: 31.95 ± 5.56% (A)
							βTCP + rhPDGF‐BB: 46.31 ± 9.06% (B)
					Group 3: βTCP + BM‐MSC (*n* = 6)		βTCP + BM‐MSC: 50.88 ± 6.68% (B)
							βTCP + rhPDGF‐BB + BM‐MSC: 72.51 ± 10.98% (C)
					Group 4: βTCP + rhPDGF‐BB + BM‐MSC (*n* = 6)		New bone formation (%) in the midportion of the defect
							βTCP: 19.10 ± 6.63% (A)
							βTCP + rhPDGF‐BB: 32.50 ± 6.09% (AB)
							βTCP + BM‐MSC: 35.74 ± 7.18% (CB)
							βTCP + rhPDGF‐BB + BM‐MSC: 48.73 ± 9.48% (C)
Hao et al. (2014b)	Xenogenous human UCMSC (*n* = 1). Negative for CD14, CD31, CD34, CD45 Positive for STRO‐1, CD29, CD44, CD73, CD90, CD105, CD166. Undifferentiated at application	8 adult male beagle dogs (48 defects)	Surgically created three‐wall intrabony defects at the mesial aspect of implants (4 × 4 × 3.5 mm)	P2‐P4 peri‐implant sites.	Group 1: PRF (*n* = 4/time point)	2, 4, and 8 weeks	BIC (%) in the defect area at
					Group 2: PRF + UCMSC (*n* = 4/time point)		2 weeks
							PRF: 17.5% (A)
							PRF + UCMSC: 20.3% (A)
							4 weeks
							PRF: 40.8% (A)
							PRF + UCMSC: 56.5% (B)
							8 weeks
							PRF: 61.2% (A)
							PRF + UCMSC: 76.2% (B)
							New bone formation (%)in the defect area at
							2 weeks
							PRF: 14.3% (A)
							PRF + UCMSC: 17.4% (B)
							4 weeks
							PRF: 37.1% (A)
							PRF + UCMSC: 49.8% (B)
							8 weeks
							PRF: 58.8% (A)
							PRF + UCMSC: 67.2% (B)
Park et al. (2014)	Autogenous PDLSC (*n* = 6). Colony‐forming efficiency, cell morphology and adherence, positive for STRO‐1 and CD146. Undifferentiated at application	6 adult male beagle dogs (24 defects)	Ligature‐induced peri‐implantitis (~40% bone loss)	P3 e P4 implant sites	Group 1: HA + collagen gel + resorbable membrane (*n* = 8)	3 months	New bone formation in the defect area
					Group 2: HA + collagen gel + PDLSC + resorbable membrane (*n* = 8)		HA + collagen gel + membrane: 1.65 ± 0.58 mm^2^ (A)
							HA + collagen gel + PDLSC + membrane: 2.10 ± 1.32 mm^2^ (A)
							HA + collagen gel + PDLSC/BMP‐2 + membrane: 4.84 ± 2.02 mm^2^ (B)
							Ratio of re‐osseointegration height to defect depth
							HA + collagen gel + membrane: 12.26 ± 6.39% (A)
							HA + collagen gel + PDLSC + membrane: 18.82 ± 11.32% (A)
							HA + collagen gel + PDLSC/BMP‐2 + membrane: 61.02 ± 27.70% (B)
					Group 3: HA + collagen gel + PDLSC/BMP‐2 + resorbable membrane (*n* = 8)		First BIC height (measured from implant apex to the most coronal BIC)
							HA + collagen gel + membrane: 5.14 ± 0.68 mm (A)
							HA + collagen gel + PDLSC + membrane: 5.11 ± 0.98 mm (A)
							HA + collagen gel + PDLSC/BMP‐2 + membrane: 6.77 ± 1.35 mm (B)
							No differences among groups were found for BIC% in the defect area and alveolar crest height (*P* > 0.2)
Yun et al. (2014)	Xenogenous human BM‐MSC (*n* = 1). Positive for CD44, CD73, CD90, and CD105. Negative for CD14, CD34, CD45, and HLA‐DR. Ability to undergo osteogenic differentiation. Undifferentiated at application	4 adult male mixed‐breed dogs (32 defects)	Surgically created three‐wall intrabony defects at the mesial aspect of osteotomy sites (4 × 4 × 4 mm)	P1‐M1 osteotomy sites	Group 1: HA (*n* = 4/time point)	6 and 12 weeks	Bone density (%) between the third and seventh threads of the implants at
							6 weeks
					Group 2: HA + BM‐MSC (*n* = 4/time point)		HA: 48.3% (A)
							HA + BM‐MSC: 39.1% (A)
					Group 3: HA + PRP (*n* = 4)		HA + PRP: 56.6% (A)
							HA + BM‐MSC + PRP: 62.2% (A)
					Group 4: HA + BM‐MSC + PRP (*n* = 4/time point)		12 weeks
							HA: 53.5% (A)
							HA + BM‐MSC: 42.5% (A)
							HA + PRP: 57.5% (A)
							HA + BM‐MSC + PRP: 72.4% (A)
							BIC (%) between the third and seventh threads of the implants at
							6 weeks
							HA: 26.3% (A)
							HA + BM‐MSC: 32.2% (A)
							HA + PRP: 19.2% (A)
							HA + BM‐MSC + PRP: 22.1% (A)
							12 weeks
							HA: 32.8% (A)
							HA + BM‐MSC: 27.2% (A)
							HA + PRP: 41.4% (A)
							HA + BM‐MSC + PRP: 42.1% (A)
Han et al. (2013)	Autogenous P‐BMPC (*n* = 4). Positive for vimentin expression, fibroblast‐like morphology, and cell adherence. Ability undergo osteogenic and adipogenic differentiation. Differentiated into osteogenic lineage at application	4 adult male mixed‐breed dogs (24 defects)	Surgically created defects at the distal aspect of osteotomy sites (6 mm H, 4 mm BL, and 5 mm MD)	P2‐ P4 osteotomy sites.	Group 1: resorbable membrane (*n* = 8)	3 months	New bone formation (%)in the defect area
							Membrane: 12.12 ± 3.08% (A)
					Group 2: injectable bone cement + resorbable membrane (*n* = 8)		Injectable bone cement + membrane: 28.02 ± 7.48% (A)
							Injectable bone cement + BMPC + membrane: 61.74 ± 3.6% (B)
							BIC (%)in the defect area
							Membrane: 18.27 ± 2.15% (A)
							Injectable bone cement + membrane: 33.13 ± 7.29% (A)
					Group 3: injectable bone cement + BMPC induced by osteogenic medium + resorbable membrane (*n* = 8)		Injectable bone cement + BMPC + membrane: 65.03 ± 3.13% (B)
Ribeiro et al. (2012)	Autogenous iliac crest BM‐MDC (*n* = 8). Cellular morphology, spreading, and adhesion. Ability to undergo osteogenic differentiation. Differentiated into osteogenic lineage at application	8 adult male beagle dogs (24 defects)	Surgically created dehiscence defects (4 × 5mm)	P3 e P4 implant sites	Group 1: no treatment (*n* = 8)	3 months	Bone fill (%)in the defect area
							No treatment: 9.96 ± 13.38% (A)
					Group 2: osteogenic differentiated BM‐MDC loaded into collagen scaffolds (*n* = 8)		BM‐MDC loaded into collagen scaffolds: 35.47 ± 20.75% (B)
							BM‐MDC loaded into collagen scaffolds + membrane: 38.66 ± 12.08% (B)
							BIC (%)in the defect area
					Group 3: osteogenic differentiated BM‐MDC loaded into collagen scaffolds + titanium reinforced ePTFE membrane (*n* = 8)		
							No treatment: 12.40 ± 13.93% (A)
							BM‐MDC loaded into collagen scaffolds: 25.39 ± 15.40% (AB)
							BM‐MDC loaded into collagen scaffolds + membrane: 32.94 ± 9.48% (B)
Zou et al. (2012)	Autogenous iliac crest BM‐MSC (*n* = 5). High CD90 and CD105 expression, low CD31 and CD34 expression. Undifferentiated at application	5 adult male Labrador dogs (30 defects)	Surgically created bone defects at the mesial aspect of the mesial extraction sockets (5 × 5 × 5 mm)	P1‐P4 mesial extraction sockets	Group 1: no treatment (*n* = 6)	3 months	(a) Histometric analysis
							Bone apposition (%) in the midportion of the defect
					Group 2: CMPC (*n* = 6)		No treatment: 1.83 ± 1.12% (A)
					Group 3: CMPC + BM‐MSC + Lenti‐GFP (*n* = 6)		CMPC: 5.42 ± 0.12% (A)
							CMPC + BM‐MSC + Lenti‐GFP: 5.65 ± 0.13% (A)
							CMPC + BM‐MSC + Lenti‐HIF: 12.78 ± 0.11% (B)
					Group 4: CMPC + BM‐MSC + Lenti‐HIF (*n* = 6)		CMPC + BM‐MSC + Lenti‐cHIF: 16.02 ± 0.32% (B)
							BIC (%) in the midportion of the defect
							No treatment: 40.06 ± 1.88% (A)
					Group 5: CMPC + BM‐MSC + Lenti‐cHIF (*n* = 6)		CMPC: 38.96 ± 4.87% (A)
							CMPC + BM‐MSC + Lenti‐GFP: 62.94 ± 6.62% (A)
							CMPC + BM‐MSC + Lenti‐HIF: 83.57 ± 2.33% (B)
							CMPC + BM‐MSC + Lenti‐cHIF: 91.24 ± 2.12% (B)
							(b) Micro‐CT analysis
							Bone fill, bone mineral density, trabecular thickness and volume fraction were significantly greater in defects treated with BM‐MSC + Lenti‐HIF or Lenti‐cHIF
Wang et al. (2011)	Autogenous iliac crest BM‐MSC (*n* = 5). Spindle shaped and ability to undergo osteogenic differentiation. Differentiated into osteogenic lineage at application	5 adult male beagle dogs (36 defects)	Surgically created, supra alveolar, peri‐implant defects (7 mm Ø × 4 mm height)	Alveolar sites	Group 1: no treatment (*n* = 6)	3 months	Mineralization apposition rate
							No treatment: 0.70 ± 0.07 mm/day (A)
					Group 2: CPC (*n* = 6)		CPC: 1.14 ± 0.06 mm/day (A)
							CPC + BM‐MSC: 1.28 ± 0.07 mm/day (A)
					Group 3: CPC + BM‐MSC (*n* = 6)		CPC + BM‐MSC + BMP‐2: 1.58 ± 0.10 mm/day (A)
							CPC + BM‐MSC + FGF: 1.43 ± 0.13 mm/day (A)
					Group 4: CPC + BM‐MSC + BMP‐2 (*n* = 6)		CPC + BM‐MSC + BMP‐2 + FGF: 1.94 ± 0.11 mm/day (B)
					Group 5: CPC + BM‐MSC + FGF (*n* = 6)		
					Group 6: CPC + BM‐MSC + BMP‐2 + FGF (*n* = 6)		
Kim et al. (2009)	Autogenous iliac crest BM‐MSC and PDLSC (*n* = 4). Colony‐forming efficiency, cellular morphology, and adherence, positive for STRO‐1 and CD146. Undifferentiated at application	4 adult male beagle dogs (24 defects)	Surgically created rectangular, saddle‐like, through and through defects (5 mm deep × 10 mm wide)	P1‐M1 alveolar sites	Group 1: HA/βTCP + resorbable membrane (*n* = 4/time point)	2 and 4 months	New bone formation (%)in the defect area at
							2 months
							HA/βTCP + membrane: 23.13% (A)
					Group 2: BM‐MSC + HA/βTCP + resorbable membrane (*n* = 4/time point)		BM‐MSC + HA/βTCP + membrane: 34.99% (B)
							PDLSC + HA/βTCP + membrane: 31.90% (B)
							4 months
							HA/βTCP + membrane: 28.36% (A)
					Group 3: PDLSC + HA/βTCP + resorbable membrane (*n* = 4/time point)		BM‐MSC + HA/βTCP + membrane: 40.17% (B)
							PDLSC + HA/βTCP + membrane: 36.51% (AB)
							BIC (%)in the defect area at:
							2 months
							HA/βTCP + membrane: ~22% (A)
							BM‐MSC + HA/βTCP + membrane: ~35% (A)
							PDLSC + HA/βTCP + membrane: ~30% (A)
							4 months
							HA/βTCP + membrane: ~18% (A)
							BM‐MSC + HA/βTCP + membrane: ~46% (B)
							PDLSC + HA/βTCP + membrane: ~28% (AB)
Ito et al. (2006)	Autogenous iliac crest BM‐MSC (*n* = unknown). Ability to undergo osteogenic differentiation. Unclear state of differentiation at application	12 adult male mixed‐breed dogs (72 defects)	Defects were surgically created using a 10 mm Ø trephine bur	P1‐M1 alveolar sites	Group 1: no treatment (*n* = 6/time point)	2 weeks, 1 and 2 months	BIC (%)at the total implant length at
							2 weeks
							No treatment: 17% (A)
							Fibrin glue + membrane: 20% (A)
					Group 2: fibrin glue + non‐resorbable membrane (*n* = 6/time point)		Fibrin glue + BM‐MSC + membrane: 22% (A)
							Fibrin glue + BM‐MSC + PRP + membrane: 25% (A)
							1 month
							No treatment: 19% (A)
							Fibrin glue + membrane: 22% (A)
					Group 3: fibrin glue + BM‐MSC + non‐resorbable membrane (*n* = 6/time point)		Fibrin glue + BM‐MSC + membrane: 32% (A)
							Fibrin glue + BM‐MSC + PRP + membrane: 49% (B)
							2 months
							No treatment: 29% (A)
							Fibrin glue + membrane: 25% (A)
					Group 4: fibrin glue + BM‐MSC + PRP + non‐resorbable membrane (*n* = 6/time point)		Fibrin glue + BM‐MSC + membrane: 42% (A)
							Fibrin glue + BM‐MSC + PRP + membrane: 53% (B)

All experimental peri‐implant defects were created on the mandible.

BIC, bone‐to‐implant contact; BL, buccolingual; BM‐MDC, bone marrow‐derived mononuclear cells; BM‐MSC, bone marrow‐derived mesenchymal stem cells; BMP‐2, bone morphogenetic protein‐2; βTCP, beta‐tricalcium phosphate; CD, cluster of differentiation; CMPC, calcium–magnesium phosphate cement; CPC, calcium phosphate cement; ePTFE, expanded polytetrafluoroethylene; FGF, fibroblast growth factor; GBR, guided bone regeneration; H, height; HA, hydroxyapatite; HLA‐DR, major histocompatibility complex, class II, DR alpha; Lenti‐GFP, lentivirus green fluorescent protein; Lenti‐cHIF, constitutively active form of lentivirus hypoxia‐inducible factor‐1α; Lenti‐HIF, lentivirus hypoxia‐inducible factor‐1α; M, molar; MD, mesio‐distal; P, premolar; P‐BMPC, peripheral blood‐acquired mesenchymal progenitor cells; PDLSC, periodontal ligament‐derived stem cells; PRF, platelet‐rich fibrin; PRP, platelet‐rich plasma; rhPDGF‐BB, recombinant human platelet‐derived growth factor; UCMSC, umbilical cord mesenchymal stem cells.

Most of the studies (nine out of 10) reported on the primary outcome selected: BIC. (Han et al. 2013; Hao et al. 2014b; Ito et al. 2006; Kim et al. 2009; Park et al. 2014; Ribeiro et al. 2012; Xu et al. 2015; Yun et al. 2014; Zou et al. 2012). Multiple secondary quantitative histological parameters were also used, including new bone formation (Hao et al. 2014b; Kim et al. 2009; Park et al. 2014; Ribeiro et al. 2012; Xu et al. 2015), bone density (Han et al. 2013; Yun et al. 2014; Zou et al. 2012), bone height (Hao et al. 2014b; Ribeiro et al. 2012), first BIC height (Park et al. 2014), ratio of re‐osseointegrated bone height (Park et al. 2014), bone fill (Ribeiro et al. 2012), and bone width (Ribeiro et al. 2012). Furthermore, few studies evaluated bone mineral apposition rates using double or triple fluorochromes (Wang et al. 2011; Zou et al. 2012). Only in one study micro‐computed tomography (CT) findings were reported, including bone fill, bone density, trabecular volume, and trabecular thickness (Zou et al. 2012). Because of the high degree of methodological heterogeneity among the included studies (defect morphology, cell source and phenotype, healing time, numbers of experimental groups, types of control groups, and methods for BIC evaluation), no meta‐analysis was performed.

### Research methods and experimental model

#### Experimental animals

Dogs were the only animal model used to study the efficacy of MSC on the healing of peri‐implant defects. All studies examined a single species in small groups of four to eight animals, all males, with age ranging from 1 to 2 years old.

#### Experimental models

Only one study induced peri‐implant inflammation to produce bone defects around implants (Park et al. 2014). In contrast, in all other studies, bone defects were surgically created at implant installation (Han et al. 2013; Hao et al. 2014b; Ito et al. 2006; Kim et al. 2009; Ribeiro et al. 2012; Wang et al. 2011; Xu et al. 2015; Yun et al. 2014; Zou et al. 2012). Differences in defect morphology were identified among the included studies that evaluated supra‐alveolar (Kim et al. 2009; Park et al. 2014; Wang et al. 2011), dehiscence (Ribeiro et al. 2012), and three‐wall intrabony defects (Han et al. 2013; Hao et al. 2014b; Xu et al. 2015; Yun et al. 2014; Zou et al. 2012). In one study, defect morphology was not clearly described (Ito et al. 2006).

In all studies, implants were left submerged during healing (Han et al. 2013; Hao et al. 2014b; Ito et al. 2006; Kim et al. 2009; Park et al. 2014; Ribeiro et al. 2012; Wang et al. 2011; Xu et al. 2015; Yun et al. 2014; Zou et al. 2012).

#### Mesenchymal stem cells

Both xenogenous (human) (Hao et al. 2014b; Yun et al. 2014) and autogenous MSC (Han et al. 2013; Ito et al. 2006; Kim et al. 2009; Park et al. 2014; Ribeiro et al. 2012; Wang et al. 2011; Xu et al. 2015; Zou et al. 2012) were used to treat peri‐implant defects. These encompassed five different types/sources of stem cells. Among these, bone marrow‐derived MSC were the most commonly used cells and were utilized in six trials (Ito et al. 2006; Kim et al. 2009; Wang et al. 2011; Xu et al. 2015; Yun et al. 2014; Zou et al. 2012). Periodontal ligament‐derived MSC were used in two studies (Kim et al. 2009; Park et al. 2014). Finally, bone marrow‐derived mononuclear cells (Ribeiro et al. 2012), peripheral blood mononuclear cells (Han et al. 2013), and umbilical cord MSC (Hao et al. 2014b) were used in only one study each. Of interest, detailed phenotypic and functional MSC characterization were provided in only one study (Yun et al. 2014). Moreover, in three trials, MSC underwent *in vitro* osteogenic differentiation before being applied into peri‐implant defects (Han et al. 2013; Ribeiro et al. 2012; Wang et al. 2011).

#### Scaffolds

Except for the study by Hao and coworkers (2014b), all studies used scaffolds to facilitate MSC application into the defects and temporarily support the structure framework.

##### Effect of MSC on the healing potential of bone defects around dental implants

Reports on the use of undifferentiated bone marrow‐derived MSC for the treatment of three‐wall peri‐implant defects yielded conflicting results. According to Xu et al. (2015), bone marrow‐derived MSC significantly increased new bone formation and BIC values as compared with beta‐tricalcium phosphate (βTCP) alone (new bone formation: BM‐MSC/βTCP = 35.74% vs. βTCP = 19.10%; *P* < 0.05) (BIC%: BM‐MSC/βTCP = 50.88% vs. βTCP = 31.95%; *P* < 0.05) (Xu et al. 2015). Conversely, in other studies, bone marrow‐derived MSC failed to improve tomographic outcomes (i.e., bone fill, bone density, and trabecular bone) (Zou et al. 2012) and histological parameters (i.e., BIC, bone density, and mineralization rate) at peri‐implant defects (Yun et al. 2014; Zou et al. 2012).

Treatment of peri‐implant defects with bone marrow‐derived MSC and bone marrow mononuclear cells that had undergone *ex vivo* osteogenic differentiation prior to clinical use (Ribeiro et al. 2012; Wang et al. 2011) resulted in higher new bone apposition than scaffolds alone. More specifically, osteo‐differentiated bone marrow‐derived MSC outstripped its scaffold regarding mineralization apposition rate by 0.14 mm/day (*P* < 0.05) (Wang et al. 2011). Likewise, osteo‐differentiated bone marrow‐derived mononuclear cells promoted superior bone fill within implant threads as compared with collagen carrier alone (35.47% vs. 9.96%, respectively; *P* = 0.0062), even though they had no effects on BIC values and bone height (Ribeiro et al. 2012).

##### The combined effect of biologically active molecules

Biomaterials used in association with stem cells included bone morphogenetic protein‐2 (BMP‐2) (Park et al. 2014; Wang et al. 2011), fibroblast growth factor (FGF) (Wang et al. 2011), platelet‐derived growth factor (PDGF) (Xu et al. 2015), hypoxia‐inducible factor‐1α (HIF) (Zou et al. 2012), platelet‐rich plasma (PRP) (Ito et al. 2006; Yun et al. 2014), and platelet‐rich fibrin (PRF) (Hao et al. 2014b).

Bone morphogenetic protein‐2 exerted an additive effect on bone regeneration in peri‐implant defects treated with MSC. Precisely, the adjunctive use of 100 ng/ml of BMP‐2 promoted greater mineralization apposition rate in peri‐implant defects treated with bone marrow‐derived MSC (BM‐MSC/BMP‐2 = 1.58 mm/day vs. BM‐MSC = 1.28 mm/day; *P* < 0.01) (Wang et al. 2011). Moreover, according to Park and coworkers (2014), periodontal ligament‐derived MSC transduced with adenoviral vectors containing BMP‐2 (BMP‐2/PDLSCs) promoted a 62% increase in bone formation within peri‐implant defects, as compared with defects treated with non‐modified periodontal ligament‐derived MSC (*P* = 0.002). This increase was accompanied by significant gains in the rate of osseointegration (*P* < 0.001), re‐osseointegration height (*P* < 0.001), and BIC height (*P* = 0.002) (Park et al. 2014).

Similarly, basic FGF (bFGF) substantially increased bone formation in peri‐implant defects treated with bone marrow‐derived MSC (BM‐MSC/bFGF = 1.43 mm/day vs. BM‐MSC = 1.28 mm/day; *P* < 0.05), albeit in lesser extent than BMP‐2 (BM‐MSC/BMP‐2 = 1.58 mm/day) (Wang et al. 2011). Finally, a combination of BMP‐2 and bFGF was more effective than either one alone in enhancing MSC‐based regeneration of bone defects around dental implants (BM‐MSC/bFGF/BMP‐2 = 1.94 mm/day) (Wang et al. 2011). Unfortunately, the impact of the adjunctive use of MSC on BMP‐2 and bFGF mediated peri‐implant bone healing has not yet been investigated.

The adjunctive use of PDGF‐BB promoted an increase in BIC values from 51% to 73%, in peri‐implant defects treated with bone marrow‐derived MSC (*P* < 0.05), without enhancing overall bone fill (Xu et al. 2015). Of interest, both new bone formation and BIC values were enhanced by the local additive delivery of bone marrow‐derived MSC in PDGF‐BB treated peri‐implant defects (new bone formation: BM‐MSC/PDGF‐BB = 49% vs. PDGF‐BB = 33%; *P* < 0.05) (BIC: BM‐MSC/PDGF‐BB = 73% vs. PDGF‐BB = 46%; *P* < 0.05) (Xu et al. 2015).

Finally, transduction of bone marrow‐derived MSC with a constitutively active truncated allele of HIF‐1α (cHIF) or a transient wild‐type HIF‐1α (tHIF)‐enhanced peri‐implant bone healing as measured by micro‐CT and histometric analysis (Zou et al. 2012). Histometric data revealed that both transient and constitutive HIF expressions enhanced by approximately twofold the healing potential of bone marrow‐derived MSC in peri‐implant bone defects, as measured by mineralization apposition rate (*P* < 0.05), bone density (*P* < 0.01), and BIC values (*P* < 0.01). Micro‐CT images showed that cHIF and tHIF bone marrow‐derived MSC increased defect fill by 59% (*P* < 0.01) and 45% (*P* < 0.01), respectively, as compared with bone marrow‐derived MSC transfected with empty vectors. Likewise, bone density was increased by 50% (*P* < 0.01) and 30% (*P* < 0.01) with the constitutive and the transient expressions of HIF, respectively. Finally, HIF expression (cHIF and tHIF) also promoted increases in trabecular bone volume (*P* < 0.01) and thickness (*P* < 0.01).

A combination of MSC and platelet concentrates has also been tested for the treatment of peri‐implant bone defects. In a large animal study (Ito et al. 2006), PRP significantly improved BIC values in peri‐implant defects treated with bone marrow‐derived MSC, from 42% to 53% (*P* < 0.05). In sharp contrast, Yun and coworkers (2014) reported that although the adjunctive use of PRP resulted in deposition of a more mature bone in peri‐implant defects treated with xenogeneic bone marrow‐derived MSC, it failed to further augment bone density and BIC values (bone density: BM‐MSC/PRP = 72.4 ± 4.7% vs. BM‐MSC = 42.5 ± 24.3) (BIC%: BM‐MSC/PRP = 42.1 ± 30.5% vs. BM‐MSC = 27.2 ± 20.2) (Yun et al. 2014). The same group also showed that the adjunctive use of xenogeneic bone marrow‐derived MSC did not enhance bone density and BIC values obtained by PRP alone (bone density: BM‐MSC/PRP = 72.4 ± 4.7% vs. PRP = 57.5 ± 22.3) (BIC%: BM‐MSC/PRP = 42.1 ± 30.5% vs. PRP = 41.4 ± 23.5) (Yun et al. 2014).

More recently, umbilical cord MSC have been shown to accelerate bone formation and stimulate greater defect fill in peri‐implant bone defects treated with a second‐generation platelet concentrate (Hao et al. 2014b). The percentage of bone fill was 58% and 67% in defects around implants treated with PRF only and PRF in association with MSC, respectively (*P* < 0.05) (Hao et al. 2014b). Finally, the addition of umbilical cord MSC to PRF significantly boosted BIC values in the defect area from 61% to 73% (*P* < 0.05) (Hao et al. 2014b).

##### The combined effect of barrier membranes

Both resorbable (Han et al. 2013; Kim et al. 2009; Park et al. 2014) and non‐resorbable (Ito et al. 2006; Ribeiro et al. 2012) membranes were commonly used in association with MSC for the treatment of peri‐implant defects. Although no study draw a direct comparison between these membrane types, post‐operative complications, such as membrane exposure, were only reported in studies using non‐resorbable membranes.

The adjunctive use of autogenous bone marrow‐derived MSC (Kim et al. 2009) and peripheral blood‐derived mesenchymal progenitor cells (Han et al. 2013) boosted the healing potential of peri‐implant defects treated with resorbable collagen membranes. The additive use of these cells not only enhanced bone formation from 28% to 40% (Kim et al. 2009) but also doubled bone density (Han et al. 2013) and BIC values within peri‐implant defects (Han et al. 2013; Kim et al. 2009). In sharp contrast, Ito et al. (2006) reported that the adjunctive use of xenogenous bone marrow‐derived MSC failed to promote increased BIC along the entire length of the implant, when used in association with non‐resorbable membranes (Ito et al. 2006).

In contrast to the trend observed with the additive use of autogenous bone marrow MSC, the adjunctive use of autogenous periodontal ligament‐derived MSC failed to improve new bone formation, BIC% (Kim et al. 2009; Park et al. 2014), and re‐osseointegration height (Park et al. 2014) in peri‐implant defects treated with resorbable membranes.

Finally, Ribeiro and coworkers (2012) demonstrated that titanium reinforced expanded polytetrafluoroethylene membranes may improve the healing potential of peri‐implant defects treated with bone marrow mononuclear cells. More specifically, the adjunctive use of non‐resorbable membranes in peri‐implant defects treated with bone marrow mononuclear cells promoted an increase in new bone formation from 1020 to 3170 mm^2^ (Ribeiro et al. 2012).

### Safety

Seven studies reported that peri‐implant sites healed uneventfully, and animals remained in good health throughout the study (Hao et al. 2014b; Ito et al. 2006; Kim et al. 2009; Park et al. 2014; Ribeiro et al. 2012; Wang et al. 2011; Yun et al. 2014). The remaining ones failed to provide information on adverse effects or signs of infection during experimental period (Han et al. 2013; Xu et al. 2015; Zou et al. 2012).

### Quality assessment of included studies

The risk of bias of included studies was assessed and listed in Table 3. Sample size calculation was unclear in all studies, despite its importance on testing new therapies, even in animal trials (Faggion et al. 2011). Randomization was accurately reported only in one study (Ribeiro et al. 2012) that used a computer‐generated sequence for randomization. The adequacy of the allocation concealment was judged as unclear in all 10 studies. Likewise, blinding of operators was not reported. Outcome assessors were reported to be blinded only in two studies (Han et al. 2013; Ribeiro et al. 2012).

**Table 3:** Risk of bias in individual studies.

Study	Randomization	Allocation concealment	Blinding of the surgeon	Blinding of the outcome assessor	Incomplete outcome data	Selective outcome reporting	Other source of bias	Overall risk of bias
Xu et al. (2015)	Unclear (no information provided)	Unclear (no information provided)	Unclear (no information provided)	Unclear (no information provided)	High (unclear if all animals and defects were evaluated at the completion of the follow‐up)	Low	Unclear (no sample size calculation)	High
Hao et al. (2014b)	Unclear (no information provided)	Unclear (no information provided)	Unclear (no information provided)	Unclear (no information provided)	High (unclear if all defects were evaluated at the completion of the follow‐up)	Low	High (unclear how 48 defects in eight animals were equally divided at 3 observing time points, given that no indication is given that the surgeries were performed at different moments)	High
Park et al. (2014)	Unclear (no information provided)	Unclear (no information provided)	unclear (no information provided)	unclear (no information provided)	Low	High (not all of the study's prespecified primary outcomes have been reported)	Unclear (no sample size calculation)	High
Yun et al. (2014)	Unclear (no information provided)	Unclear (no information provided)	Unclear (no information provided)	Unclear (no information provided)	Low	Low	Unclear (no sample size calculation)	Unclear
Han et al. (2013)	Unclear “each defect was randomly assigned”	Unclear (no information provided)	Unclear (no information provided)	Low	Low	Low	Unclear (no sample size calculation)	Unclear
Ribeiro et al. (2012)	Low “randomization was performed according to a computer‐generated code”	Unclear (no information provided)	Unclear (no information provided)	Low	Low	Low	Unclear (no sample size calculation)	Unclear
Zou et al. (2012)	Unclear “defects were generated and randomly allocated”	Unclear (no information provided)	Unclear (no information provided)	Unclear (no information provided)	Unclear (number of defects analyzed at the completion of the follow‐up interval not clearly stated)	Low	Unclear (no sample size calculation)	Unclear
Wang et al. (2011)	Unclear (no information provided)	Unclear (no information provided)	Unclear (no information provided)	Unclear (no information provided)	Low	Low	Unclear (no sample size calculation)	Unclear
Kim et al. (2009)	Unclear “were randomly assigned to the three prepared defects”	Unclear (no information provided)	Unclear (no information provided)	Unclear (no information provided)	Low	Low	Unclear (no sample size calculation)	Unclear
Ito et al. (2006)	Unclear “selection of the treatments and localization was random”	Unclear (no information provided)	Unclear (no information provided)	Unclear (no information provided)	Unclear (number of defects excluded from the final analysis not stated)	Low	Unclear (no sample size calculation; unclear description of the defect model)	Unclear

All of the included studies reported the primary outcome as BIC (Han et al. 2013; Hao et al. 2014b; Ito et al. 2006; Kim et al. 2009; Park et al. 2014; Ribeiro et al. 2012; Xu et al. 2015; Yun et al. 2014; Zou et al. 2012) or bone mineral apposition rate (Wang et al. 2011). They all appeared to be free of selective reporting with respect to the primary outcome. In seven of the included studies, all peri‐implant defects randomized were included in the final analysis (Han et al. 2013; Hao et al. 2014b; Kim et al. 2009; Park et al. 2014; Wang et al. 2011; Xu et al. 2015; Yun et al. 2014). In two trials, some peri‐implant defects were excluded from the analysis following randomization, and data were analyzed by protocol (Ito et al. 2006; Ribeiro et al. 2012). In the study by Ribeiro et al. (2012), two out of the eight defects treated with a combination of titanium reinforced expanded polytetrafluoroethylene membranes and bone marrow‐derived MSC were lost during follow‐up because of membrane exposure. Although Ito et al. (2006) stated that defects were also excluded because of membrane exposure during healing, the number of defects excluded was not reported. Finally, Zou et al. (2012) failed to report if all defects randomized were included in the final analysis. In one study (Ito et al. 2006), defect morphology was not clearly described.

Taken together, three studies were considered at overall “high risk” of bias (Hao et al. 2014b; Park et al. 2014; Xu et al. 2015), and seven were considered at overall “unclear risk” of bias (Han et al. 2013; Ito et al. 2006; Kim et al. 2009; Ribeiro et al. 2012; Wang et al. 2011; Yun et al. 2014; Zou et al. 2012).

## Discussion

Development of novel strategies to promote predictable bone neoformation/regeneration around dental implants is of clinical interest. Along this line, this systematic review provides a comprehensive assessment of the effects of distinct populations of MSC in the healing of peri‐implant bone defects in large animal models.

We found that undifferentiated bone marrow‐derived MSC yielded conflicting results in the treatment of three‐wall peri‐implant defects. While Xu et al. (2015) demonstrated increased bone formation and BIC values in peri‐implant defects treated with autogenous bone marrow‐derived MSC, Yun et al. (2014) and Zou et al. (2012) failed to find differences in healing outcomes in peri‐implant defects treated with or without MSC. The basis for contrasting results is currently unknown but might be related to differences in factors known to influence the osteogenic potential of MSC, including age of the donors, tissue of MSC origin, heterogeneity of selectively isolated MSC subpopulations, MSC *ex vivo* expansion conditions, scaffold composition, and three‐dimensional arrangement (Harris and Cooper 2004; Lee et al. 2001; Shamsul et al. 2004). Additionally, these differences may be related to the use of virally transfected cells by Zou and collaborators (2012) and the use of xenogeneic MSC by Yun and coworkers (2014). Although no study directly compared the efficacy of xenogeneic and autogenous MSC in the healing of peri‐implant bone defects, xenogenic transplantation of MSCs has been shown to promote poorer bone regeneration than autologous transplantation of MSCs in tibia bone defects (Niemeyer et al. 2010).

Transplantation of *ex vivo* osteo‐differentiated autogenous bone marrow‐derived MSC and bone marrow mononuclear cells promoted increased bone apposition in peri‐implant defects (Ribeiro et al. 2012; Wang et al. 2011). Although it remains unclear if predifferentiated MSC are superior to undifferentiated cells in promoting peri‐implant bone healing, some indirect evidence of superiority of differentiated over undifferentiated MSC comes from a study that evaluated bone marrow‐derived MSC transduced with HIF, a short‐lived transcriptional activator that regulates osteogenic genes (Drager et al. 2015; Mamalis and Cochran 2013). Accordingly, transduction of bone marrow‐derived MSC with a cHIF or a tHIF enhanced by twofold the healing potential of undifferentiated bone marrow‐derived MSC in peri‐implant bone defects (Zou et al. 2012). However, in sharp contrast, in other experimental models, osteoblastic predifferentiation of MSC failed to promote increased ectopic bone formation over the one obtained with undifferentiated cells (De Kok et al. 2006). Thus, the significance of *ex vivo* MSC predifferentiation still requires confirmation.

Bone‐forming osteoblasts are non‐replicating cells derived from MSC (Park et al. 2012). Accordingly, new bone formation relays on MSC and molecular signals that favor MSC osteoblastic differentiation. Along with these lines, we found that a few growth factors, namely, BMP‐2, FGF, and PDGF, enhance the efficacy of MSC on the healing of peri‐implant defects (Park et al. 2014; Wang et al. 2011; Xu et al. 2015; Zou et al. 2012). BMP‐2 influences cellular behaviors known to affect bone and cartilage formation. Its properties are confined primarily in the early stages of bone formation and bone repair, when BMP‐2 promotes MSC differentiation to osteoblast precursors and the development of these precursors into mature osteoblasts (Carreira et al. 2014; Darby and Morris 2012; Haversath et al. 2012). FGF has crucial roles on bone repair by promoting osteoblastic differentiation and enhancing the osteoinductive activity of BMP‐2 (Du et al. 2012; Fujimura et al. 2002). Moreover, FGF has been shown to enhance MSC survival (Bianchi et al. 2003; Eiselleova et al. 2009) and MSC osteogenic differentiation (Hou et al. 2007; Tanaka et al. 2003; Tsutsumi et al. 2001). Likewise, PDGF promotes MSC osteogenic differentiation and induces vascular endothelial growth factor (VEGF) expression, thereby supporting angiogenesis during wound healing (Darby and Morris 2012; Shah et al. 2012).

Contrary to the additive effects of specific growth factors on MSC‐mediated peri‐implant bone healing, the effects of platelet concentrates are less clear. PRP consists of an aggregate of PDGFs including PDGF‐AA, PDGF‐BB, PDGF‐AB, transforming growth factor‐beta, platelet‐derived epidermal growth factor, platelet‐derived angiogenesis factor, insulin growth factor‐1, and platelet factor‐4 (van den Dolder et al. 2006). PRP is thought to support bone regeneration, presumably through the action of growth factors. However, its effects are likely to be limited by the quick, non‐sustained release of these factors and lack of BMP‐2 (Kumar & Shubhashini, 2013). Not surprisingly, Yun and coworkers (2014) reported that the adjunctive use of PRP failed to further augment bone density and BIC values in peri‐implant defects treated with undifferentiated bone marrow‐derived MSC. However, it is also conceivable that this lack of additional gains by the adjunctive use of PRP may be related to a mismatch between xenogenic MSC and autogenous PRP used by Yun and coworkers (2014). Lastly, although one study reported that PRP improves bone healing in peri‐implant defects treated with autologous bone marrow‐derived MSC (Ito et al. 2006), it is unclear if MSC used in this study were applied at the defect site in an undifferentiated or differentiated state.

In all but one study, the adjunctive use of autogenous bone marrow‐derived MSC boosted the healing potential of peri‐implant defects treated with resorbable collagen membranes (Han et al. 2013; Kim et al. 2009). It can be argued, however, that the lack of additive effect reported by the exception study (Ito et al. 2006) was because histological measurements were taken along the entire length of the implants, instead of within the defect lengths only and therefore included both newly formed and pristine bone.

Interestingly, the adjunctive use of autogenous periodontal ligament‐derived MSC failed to improve bone healing within peri‐implant defects treated with resorbable membranes (Kim et al. 2009; Park et al. 2014). Thus, it is reasonable to hypothesize that periodontal ligament‐derived MSC may not be an ideal source of MSC for the treatment of peri‐implant defects. This observation is in consensus with reports that demonstrated that although periodontal ligament‐derived MSC have the ability to form mineralized deposits, their mineralization and osteogenesis potentials are markedly lower from those reported for their bone marrow‐derived counterparts (Vasandan et al. 2014).

Periodontal ligament‐derived MSC share similarities to other MSC with respect to clonality, surface‐antigen profiles, and generation of multiple types of differentiated cells (Kim et al. 2007). However, a thorough one‐to‐one comparison of periodontal ligament‐derived and bone marrow‐derived MSC for their surface characteristics revealed key differences in the expression of mesenchymal (CD105) and pluripotent/multipotent stem cell‐associated cell surface antigens (i.e,, SSEA4, CD117, CD123, and CD29) (Vasandan et al. 2014). To this regard, the lower osteogenic potential of periodontal ligament‐derived MSC could be related to their lower levels of CD105 expression. Detailed comparative molecular studies of osteoblasts derived from primary cultures and those derived from periodontal ligament and bone marrow could potentially explain the different clinical outcomes obtained with periodontal ligament‐derived and bone marrow‐derived MSC. However, to the knowledge of the authors, these studies are yet to be published.

## Conclusions

The available preclinical controlled animal model studies that investigated the effect of MSC on peri‐implant defect bone healing are limited in number and have small sample size, exhibiting high or moderate risk of bias. Despite the quality level of the existing evidence, the existing data indicate that

- The intraoral use of MSC in the treatment of peri‐implant defects was not associated with local or systemic adverse effects in preclinical studies. However, its therapeutic safety in humans remains to be investigated.
- The use of MSC may provide beneficial effects on the bone healing within defects around dental implants. *Ex vivo* osteogenic differentiation of MSC prior to defect application appears to be advantageous.
- It is likely that the various degrees of success of MSC in peri‐implant bone healing are related to the use of distinct populations of MSC derived from multiple lineages, tissues, and donor species (i.e., autologous vs. xenogenous). Analysis of existing low‐level evidence suggests that autologous bone marrow‐derived MSC grant superior results. However, much work remains to be performed to identify phenotypic profiles of highly osteogenic MSC populations.
- The combination of MSC with barrier membranes and growth factors (i.e., BMP‐2, FGFb, PDGF‐BB, HIF, PRP, and PRF) appears to provide improved treatment outcomes.
- Human investigations are necessary to confirm if the improved histological parameters observed in large animal studies are indeed translated into clinical gains.

## Conflict of Interest

The authors have reported no conflicts of interest.
